# Effect of thermomechanical aging on force system of orthodontic aligners made of different thermoformed materials: An in vitro study

**DOI:** 10.1007/s00056-024-00527-0

**Published:** 2024-04-23

**Authors:** Tarek M. Elshazly, Ludger Keilig, Diva Nang, Bijan Golkhani, Anna Weber, Hanaa Elattar, Sameh Talaat, Christoph Bourauel

**Affiliations:** 1 Oral Technology Department, Dental School, University Hospital Bonn, Bonn, Germany; 2 Department of Dental Prosthetics, Propaedeutics and Materials Science, Dental School, University Hospital Bonn, Bonn, Germany; 3 Orthodontic Department, Faculty of Dentistry, Umm Al-Qura University, Makkah, Saudi Arabia; 4 Orthodontic Department, Faculty of Dentistry, Suez Canal University, Ismailia, Egypt; 5 Orthodontic Department, Future University in Egypt, Cairo, Egypt; 6 Oral Technology, University Hospital Bonn, Welschnonnenstr. 17, 53111 Bonn, Germany

**Keywords:** Biomechanics, Orthodontic forces, Tooth movement, Clear aligner appliances, Torque, Biomechanik, Kieferorthopädische Kräfte, Zahnbewegung, Transparente Aligner, Drehmoment

## Abstract

**Purpose:**

The aim was to investigate the effect of aging by thermocycling and mechanical loading on forces and moments generated by orthodontic clear aligners made from different thermoplastic materials.

**Methods:**

A total of 25 thermoformed aligners made from 5 different materials, i.e., Essix ACE® and Essix® PLUS™ (Dentsply Sirona, Bensheim, Germany), Invisalign® (Align Technology, San Jose, CA, USA), Duran®+ (Iserlohn, Germany), Zendura™ (Fremont, CA, USA), underwent a 14-day aging protocol involving mechanical loading (a 0.2 mm vestibular malalignment of the upper left second premolar [tooth 25]) and thermocycling in deionized water (temperature range 5–55 °C). The 3D forces/moments exerted on tooth 25 of a resin model were measured at three time points: before aging (day 0), after 2 days and after 14 days of aging.

**Results:**

Before aging, extrusion–intrusion forces were 0.6–3.0 N, orovestibular forces were 1.7–2.3 N, and moments as mesiodistal rotation were 0.3–42.1 Nmm. In all directions, multilayer Invisalign® exhibited the lowest force/moment magnitudes. After aging, all materials showed a significant force/moment decay within the first 2 days, except Invisalign® for orovestibular and vertical translation. However, following thermomechanical aging, Duran®+ and Zendura™ aligners had equivalent or even higher vestibular forces (direction of mechanical load).

**Conclusion:**

Thermomechanical aging significantly reduced forces and moments during the first 48 h. Multilayer aligner materials exhibit lower initial forces and moments than single-layer ones, and were less influenced by aging. Material hardening was observed after subjecting some of the aligner materials to mechanical loading. Thus, orthodontists should be aware of possible deterioration of orthodontic aligners over time. This work also sheds light on how material selection impacts the mechanical behavior of aligners and may provide valuable guidance regarding optimal timing for the aligner changing protocol.

## Abstract (de)

**Ziel:**

Ziel war es, die Auswirkung der Alterung durch Thermocycling und mechanische Belastung auf von kieferorthopädischen Alignern aus verschiedenen thermoplastischen Materialien erzeugte Kräfte und Drehmomente zu untersuchen.

**Methoden:**

Insgesamt 25 tiefgezogene Aligner aus 5 verschiedenen Materialien, Essix ACE® und Essix® PLUS™ (Dentsply Sirona, Bensheim, Deutschland), Invisalign® (Align Technology, San José/CA, USA), Duran®+ (Iserlohn, Deutschland), Zendura™ (Fremont/CA, USA), wurden einem 14-tägigen Alterungsprotokoll unterzogen, das eine mechanische Belastung (eine 0,2 mm vestibuläre Fehlstellung des oberen linken zweiten Prämolaren [Zahn 25]) und Thermocycling in demineralisiertem Wasser (Temperaturbereich 5–55 °C) umfasste. Die auf den Zahn 25 eines Kunststoffmodells ausgeübten 3‑D-Kräfte/-Drehmomente wurden zu 3 Messzeitpunkten ermittelt: vor der Alterung (Tag 0) sowie 2 und 14 Tage nach der Alterung.

**Ergebnisse:**

Vor der Alterung betrugen die Extrusion-Intrusion-Kräfte 0,6–3,0 N, die orovestibulären Kräfte 1,7–2,3 N und die Drehmomente als mesiodistale Rotation 0,3–42,1 Nmm. In allen Richtungen wies das mehrschichtige Invisalign® die niedrigsten Kraft‑/Drehmomente auf. Nach der Alterung zeigten alle Materialien innerhalb der ersten 2 Tage einen signifikanten Kraft/Drehmomente-Abfall, außer Invisalign® für die orovestibuläre und vertikale Translation. Nach der thermomechanischen Alterung wiesen Duran®+- und Zendura™-Aligner jedoch gleichwertige oder sogar höhere vestibuläre Kräfte auf (Richtung der mechanischen Belastung).

**Schlussfolgerung:**

Die thermomechanische Alterung reduzierte die Kräfte und Drehmomente in den ersten 48 h erheblich. Mehrschichtige Aligner-Materialien wiesen geringere Anfangskräfte und -momente auf als einschichtige und wurden durch die Alterung weniger beeinflusst. Nach der mechanischen Belastung einiger Aligner-Materialien wurde eine Materialverhärtung beobachtet. Kieferorthopäden sollten sich daher über eine mögliche Verschlechterung der Aligner im Laufe der Zeit im Klaren sein. Diese Arbeit wirft auch ein Licht darauf, wie sich die Materialauswahl auf das mechanische Verhalten von Alignern auswirkt, und kann wesentliche Hinweise auf den optimalen Zeitpunkt für das Aligner-Wechselprotokoll liefern.

## Introduction

Orthodontic clear aligners offer a promising and increasingly popular alternative to the traditional wire/bracket system, although there is an ongoing debate regarding their effectiveness [1]. They provide a treatment solution that is esthetically superior, more hygienic, and comfortable [2]. However, achieving optimal clinical outcomes relies on the meticulous optimization of several factors [3, 4]. One such crucial factor is the selection of the appropriate aligner material, since material behavior strongly influences the performance of the aligners. In orthodontic applications, the ideal scenario necessitates materials that can apply continuous light forces over an extended period [4–6]. Furthermore, aligner materials should revert to their original shape after being removed from the oral cavity. This behavior requires a material holding a suitable level of stiffness to apply the required light force, coupled with a high elastic limit to prevent permanent deformation [7].

Nowadays, the most commonly employed technique for manufacturing aligners involves thermoforming of thermoplastic sheets. These sheets are composed of various viscoelastic polymers, include ethylene vinyl acetate (EVA), polypropylene (PP), polystyrene (PS), polyethylene terephthalate (PET), polyethylene terephthalate glycol (PET-G), thermoplastic polyurethane (TPU), or polycarbonate (PC) [8, 9]. Viscoelastic materials exhibit a notable phenomenon such as creep and stress relaxation, resulting in significant variations in their mechanical behavior over time under loading conditions [7, 10]. In addition, the mechanical properties of thermoformed sheets display substantial differences when compared to those of their raw counterparts [11, 12].

Patients receive instructions to wear their aligner splints throughout the day, removing them only during meals and for oral hygiene measurements. Each aligner splint is typically worn for approximately 2 weeks before being replaced by the next one [3, 13]. Accordingly, each aligner during this period of 14 days goes through intermittent thermal and mechanical loads. Thermal stresses arise from both the standard oral temperature (37 °C) and the intake of hot and cold beverages. Short-term mechanical loads arise during the insertion and the removal of the aligners, and long-term loads arise from the continuous contact between the aligners and malaligned teeth, as well as the forces exerted by occlusal contacts [3].

The existing literature is still deficient in providing a thorough understanding of aligner biomechanics. In this light, our research team is leading an extensive project on orthodontic aligners, investigating various aspects at multiple levels. Part of this project is providing valuable insights into how the mechanical properties of aligners are affected by aging over various durations by employing different aging agents [14], different aging techniques [15], different measurement methods, and different aligner materials. Using the methods developed in the previous studies, the aim of the current study was to report the change of forces and moments generated by different thermoformed aligner materials after undergoing a 14-day aging protocol involving both thermocycling and mechanical loading. A special focus was on the comparison between the different tested materials.

## Materials and methods

A three-dimensional (3D) dataset of a fully dentate maxilla (Digimation, St. Rose, LA, USA) was imported into the image processing software (3-matic 16.0; Materialise, Leuven, Belgium) in order to design two distinct models: one aligned model, and a second malaligned model where the upper left second premolar (tooth 25) underwent a facial translation of 0.2 mm. Subsequently, the digital models were 3D printed: one from the aligned model for thermoforming and five from the malaligned model for simulating mechanical aging. The printing process utilized P pro resin (Straumann, Basel, Switzerland) and a 3D printer (P20+; Straumann, Basel, Switzerland).

**Fig. 1:**
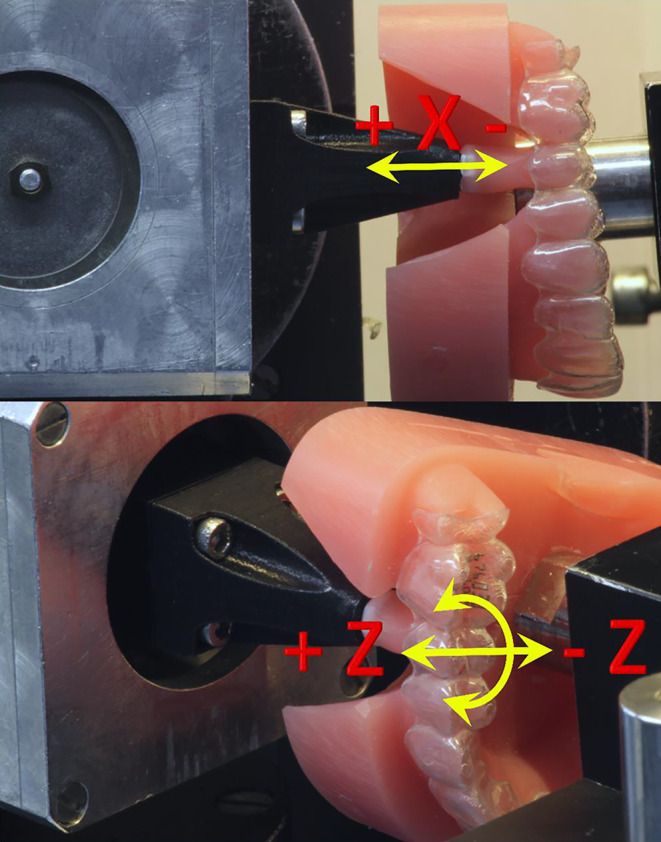
Resin model with aligner fitted into the orthodontic measurement and simulation system (OMSS). Movable tooth 25 was adjusted in the neutral position in the aligner. Simulation of three tooth movements were carried out: intrusion (−ve)/extrusion (+ve) along the X axes by 0.2 mm (upper), oral (−ve)/vestibular (+ve) translation along the Z axes by 0.2 mm, and 2° rotation around the X axis in mesial (+ve) or distal (−ve) direction (lower) Kunststoffmodell mit in das kieferorthopädische Mess- und Simulationssystem (OMSS) eingesetztem Aligner. Der bewegliche Zahn 25 wurde in der neutralen Position im Aligner eingestellt. Drei Zahnbewegungen wurden simuliert: Intrusion (‑ve)/Extrusion (+ve) entlang der X‑Achse um 0,2 mm (oben), orale (‑ve)/vestibuläre (+ve) Translation entlang der Z‑Achse um 0,2 mm und 2° Rotation um die X‑Achse in mesialer (+ve) bzw. distaler (‑ve) Richtung (unten)

In the current study, five different types of thermoplastic sheets, manufactured by four different companies having different composition, were investigated (Table 1). Using a standard thermoforming device (Ministar S®; Scheu-Dental, Iserlohn, Germany) and following the manufacturers’ instructions, five aligners (*n* = 5) were made from each material by thermoforming over the aligned model resulting in a total of 25 aligners being manufactured. The thermoformed aligners were trimmed along the gingival margin in a scalloped design (Fig. 1). All but the Invisalign® aligners were produced identically on the same model and by a skilled technician. The Invisalign® aligners were procured directly from the manufacturer, as the raw sheet material is not freely available on the market.

**Table 1:** Investigated aligner materials, including manufacturer, material thickness, and composition Untersuchte Aligner-Materialien, einschließlich Hersteller, Materialstärke und Zusammensetzung

Name	Manufacturer	Thickness	Composition
Essix ACE®	Dentsply Sirona (Bensheim, Germany)	0.75 mm	Single-layer (PET)
Essix®PLUS™	Dentsply Sirona (Bensheim, Germany)	0.80 mm	Unknown
Invisalign®	Align Technology (San Jose, CA, USA)	0.75 mm	Multilayer^a^
Duran®+	Scheu-Dental (Iserlohn, Germany)	0.75 mm	Single-layer (PET-G)
Zendura™	Bay Materials (Fremont, CA, USA)	0.76 mm	Single-layer (TPU)

*PET* polyethylene terephthalate, *PET‑G* polyethylene terephthalate glycol, *TPU* thermoplastic polyurethane

^a^ The Invisalign thermoformed aligners utilized in this investigation were procured directly from the manufacturer, lacking any explicit details regarding the fabrication conditions, sheet thickness, and composition

Following the measurement protocol already established in previous studies [14, 15], the aligners were thermocycled for 14 days to simulate a 2-week period of a wearing protocol of the aligners in the oral cavity. The two water basins of the thermocycling device (Thermocycler THE-1100; SD Mechatronik, Feldkirchen-Westerham, Germany) were filled with deionized water (Ampuwa; Fresenius Kabi, Bad Homburg, Germany). Each cycle lasted for 94 s and consisted of 87 s of storage in a 37 °C warm basin, followed by 1 s of draining time, then 1 s of storage in a 6 °C cold basin, followed by 1 s of draining time and a total dwell time of 4 s. During the thermocycling phase, the aligners were kept in position on the malaligned models. Consequently, the aligners were exposed to both thermocycling and mechanical loading due to the malalignment of tooth 25 in the models.

The force and moment measurements were recorded on day 0 (before aging), day 2 (around 1838 cycles), and day 14 (around 12,867 cycles) of thermocycling, using the OMSS ([16]; Fig. 1). The OMSS is a custom-made biomechanical device, whose measuring unit is coupled with a sensor. It can be used to replicate 3D tooth movements, while measuring force/deflection or moment/rotation ratios. For the measurement purposes, an additional resin model (Technovit 4004; Kulzer, Hanau, Germany) was created, in which tooth 25 was sawn out, and the neighboring teeth were slightly ground proximally to ensure smooth insertion of the tooth without any resistance. The sawn tooth 25 was then attached to the measuring unit, while the resin model was securely fixed. The occlusal plane of the model was set parallel to the sensor axis (Fig. 1). The tooth was adjusted to its neutral position within the dental arch, ensuring that no forces or moments were being applied to the tooth in the initial position. In separate measurements, the measuring unit with the tooth was moved in the X (intrusion/extrusion) and Z (orovestibular translation) axes by ± 0.2 mm and rotated in mesial and distal directions around the X axis by ± 2°, where positive values (+) represent extrusive and vestibular forces and mesial moments and vice versa (Fig. 1). For each measurement, force/translation and moment/rotation curves were recorded. After each measurement, the adjustment of the measurement setup was rechecked and corrected if necessary.

## Statistical analysis

Sample size calculation was performed using the software G × Power (version 3.1; Duesseldorf, Germany) [38], with a study power of 80% and a significance level of 0.05. Effect size was derived based on a study by Hahn et al. [17]. The resulting minimum sample size was four samples per group. In the current study, five samples per group were used (*n* = 5).

The primary data from each group underwent analysis to determine the maximum values of forces and moments. The normal distribution of the data was tested using the Kolmogorov–Smirnov test, and all data were found to be normally distributed. Mean values, standard deviations, and Student’s t‑test (two-sided, unpaired) for normally distributed data were employed for the analysis. To account for multiple testing, the Bonferroni correction was applied. A difference was considered significant if the *p*-value was less than 0.05. Direct pairwise comparisons were conducted to examine differences between the groups. The results were visually represented in bar graphs and tables, illustrating the changes in forces and moments over time for each group. The statistical analyses were performed using Excel (2016; Microsoft, Redmond, WA, USA) by utilizing available statistical functions as well as self-developed routines.

## Results

**Fig. 2:**
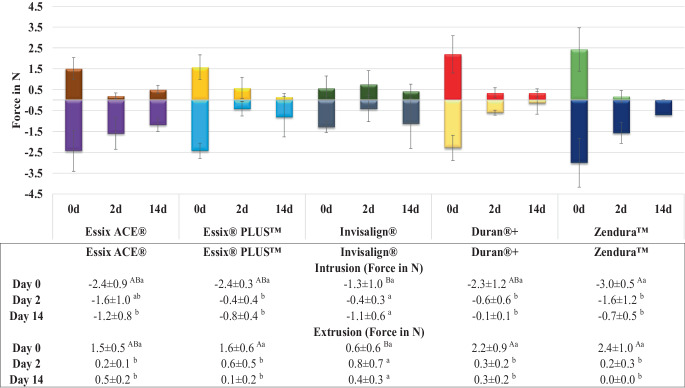
Change in mean intrusive/extrusive forces of aligners made from different thermoformed sheets exerted on tooth 25 at different time points. Different upper- and lowercase superscript letters indicate a statistically significant difference within the same horizontal row and vertical column, respectively. *d* day(s) Veränderung der mittleren Intrusions‑/Extrusionskräfte von Alignern aus verschiedenen Tiefziehfolien, die zu verschiedenen Zeitpunkten auf den Zahn 25 ausgeübt wurden. Unterschiedliche hochgestellte Groß- und Kleinbuchstaben zeigen einen statistisch signifikanten Unterschied innerhalb derselben horizontalen Zeile bzw. vertikalen Spalte an. *d *Tag(e)

As shown in Fig. 2, the mean forces for intrusion and extrusion before the aging process were measured in the range of 1.3–3.0 N and 0.6–2.4 N, respectively. Before aging, the material Zendura demonstrated the highest force values, but the measured differences were insignificant from the other single-layer aligner materials. On the other hand, the multilayer Invisalign material exhibited the significantly lowest force values. Following thermomechanical aging, all tested materials experienced a significant decrease in mean forces compared to their pre-aging levels, except for Invisalign. The significant force reduction occurred within the first 2 days, with no further significant drop observed after the 14 days (Fig. 2).

**Fig. 3:**
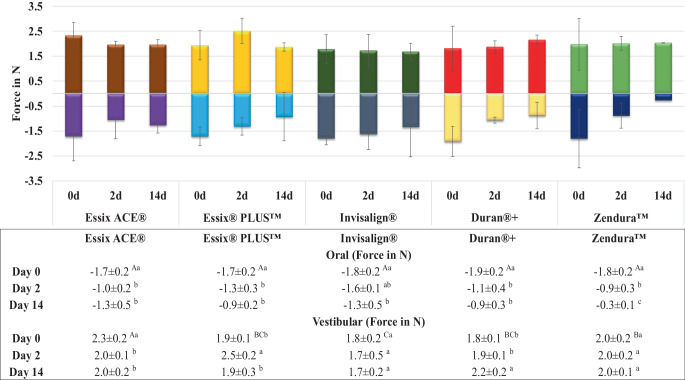
Change in mean oral/vestibular forces of aligners made from different thermoformed sheets exerted on tooth 25 at different time points. Different upper- and lowercase superscript letters indicate a statistically significant difference within the same horizontal row and vertical column, respectively. *d* day(s) Veränderung der mittleren oralen/vestibulären Kräfte von Alignern aus verschiedenen Tiefziehfolien, die zu verschiedenen Zeitpunkten auf den Zahn 25 ausgeübt wurden. Unterschiedliche hochgestellte Groß- und Kleinbuchstaben zeigen einen statistisch signifikanten Unterschied innerhalb derselben horizontalen Zeile bzw. vertikalen Spalte an. *d *Tag(e)

Figure 3 displays that the forces exerted by the aligners in the oral–vestibular direction were generally lower than those measured in the intrusion–extrusion directions. Before the aging process, the mean oral and the vestibular forces were in the range of 1.7–1.9 N and 2.3–1.8 N, respectively. There were no significant differences between all tested materials in terms of forces in the oral direction. In the vestibular direction, Essix ACE demonstrated the highest force values and Invisalign exhibited the lowest. Following 14 days of thermomechanical aging, all materials experienced a significant decrease in mean forces in the oral direction compared to their pre-aging levels. In the vestibular direction only Essix ACE showed a significant force decay. Deviating from the other materials, Duran+ demonstrated an increase in the forces after 14 days of thermomechanical loading (Fig. 3).

**Fig. 4:**
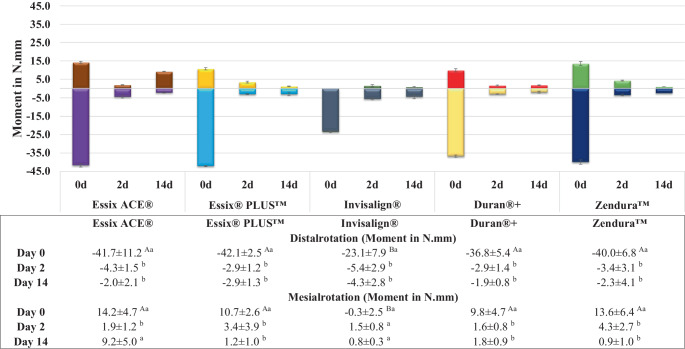
Change in mean distal/mesial rotation moment generated by aligners made from different thermoformed sheets exerted on tooth 25 at different time points. Different upper- and lowercase superscript letters indicate a statistically significant difference within the same horizontal row and vertical column, respectively. *d* day(s) Veränderung des mittleren distalen/mesialen Rotationsmoments, das von Alignern aus verschiedenen Tiefziehfolien zu verschiedenen Zeitpunkten auf den Zahn 25 ausgeübt wird. Unterschiedliche hochgestellte Groß- und Kleinbuchstaben zeigen einen statistisch signifikanten Unterschied innerhalb derselben horizontalen Zeile bzw. vertikalen Spalte an. *d *Tag(e)

In Fig. 4, the mean moment values for distal rotation ranged from 23.1 to 42.1 Nmm, while with mesial rotation, moment ranged from 0.3 to 14.2 Nmm. In both rotation directions, Invisalign demonstrated the lowest moment values which were significantly different from the other aligner materials, especially in the mesial direction. On average, the moments for distal rotation were four times larger than those for mesial rotation. Following 2 days of thermomechanical loading, all materials experienced a significant drop of the moments compared to their pre-aging levels, with no further significant drop observed after 14 days. Remarkably, Invisalign aligners exhibited poor performance in inducing mesial rotation and produced minimal moments at all time points (Fig. 4).

## Discussion

In the current study, five different conventional thermoplastic aligner materials with different chemical characteristics were employed to examine the impact of thermomechanical aging on force and moment generation during 3D movement of tooth 25. Throughout a 2-week period, the aligners were exposed to a mechanical loading induced by the vestibular malalignment of tooth 25 as well as a thermal loading by thermocycling in deionized water. To mimic a situation closer to clinical conditions, artificial saliva would have been the preferred option. However, due to technical limitations of the thermocycling device, deionized water was employed as an alternative. Moreover, a previous study already showed that artificial saliva and deionized water have similar effect in artificial aligner aging [14].

Force measurements were recorded prior to the aging process, after 2 days, and upon the termination of the 14 days of aging. This approach was adopted due to previously reported low force changes between day 2 and day 14, as it was observed in our own group [14, 15]. Other groups similarly reported considerable stress-drop in aligner materials occur during the first 8–48 h of usage [7, 18, 19], and thus the results of all further examination days may no longer differ significantly from each other.

Proffit et al. [20] gave approximate recommendations regarding the optimal force specifications for various tooth movements, i.e., 0.1–0.2 N for intrusion and 0.7–1.2 N for bodily tooth movement. Sander et al. [21] indicated as well that the suitable moment range for rotating tooth 25 was 10.5–15.8 Nmm. However, the forces and moments generated by aligners recorded in the present study as well as the values reported in many previous experimental studies exceed these recommendations. The reason behind this fact may be attributed to the lack of considering the properties of the periodontal ligament (PDL) in experimental setups which act as a cushion. Another plausible rationale could be linked to the fact that, under clinical conditions, the tooth tends to move over time leading to a reduction of the initially generated forces [22]. Moreover, forces exerted by aligners have been shown to be primarily high on initial insertion, but they subsequently diminish over time, as it was evidenced in the current study [19, 23–26].

Therefore, when assessing the effectiveness of an aligner, it is crucial to examine not only the initial insertion force but also the sustained force it applies throughout its usage duration [7].

Hahn et al. [27, 28], using a biomechanical set-up similar to the one employed in the current study, reported that the horizontal forces exerted by the aligner on upper central incisor were around 2.7 N and the intrusive forces were 0.4 N. Likewise, Elkholy et al. [29] measured 4.5 N (Duran) and 5.2 N (Invisalign®) forces on a 0.25 mm palatally deflected upper central incisor, and 42.5 Nmm average moment on a 15° distally rotated mandibular canine [30]. Kohda et al. [31] reported generated forces at 2.91 N by 0.75 mm thick Duran aligners, upon a 0.5 mm upper central incisor misalignment. Barbagallo et al. [32] measured, clinically, 5.1 N forces generated on a first premolar tilted 0.5 mm palatally using pressure sensitive sheets. Engelke et al. [24] recorded moments in the range of 4.3–20.2 Nmm on 10° rotations of upper central incisor. These findings are consistent somehow with the current study’s outcomes. Nevertheless, the discrepancies in values across studies may be attributed to variances in the experimental measurement protocol, material composition, trimming line design, extent and type of tooth activation, the type of the targeted tooth, and aligner thickness.

As reported previously [7, 19, 26], the inherent strength of aligners declines rapidly under oral conditions. Hence, for a comprehensive study of aligner behavior, it is essential to either conduct an in vivo study or replicate the clinical conditions in an in vitro setup. In the current study, thermocycling that included water storage and temperature fluctuation (between 5 and 55 °C) was conducted. When the aligner sheets are immersed in water, water molecules are absorbed into the plastic material through diffusion between the polymer chains, increasing their mobility [7, 33]. In addition, an elevation in temperature further enhances the movement of these polymer molecules, causing further weakening of the plastic material [7, 34]. Nevertheless, the sole application of thermocycling was reported to have a negligible impact on the force generation by orthodontic aligners, but the accumulating mechanical stresses due to repeated persistent mechanical loading contributed significantly to the phenomenon of force decay [15, 25]. Hence, the concurrent application of both thermal and mechanical loads, as in the present study, is thought to account for the observed decrease in force/moment within almost all investigated materials. On the other hand, sustained or even increased force generation in the vestibular direction was observed with some aligner materials, which may be primarily attributed to cold hardening of the vestibular segments of the aligner corresponding to the location of the malaligned tooth which leads to an increase in hardness [35].

The aligners from Invisalign® used in the current study were obtained directly from the manufacturer with a limited information available about their composition, except for the fact that they are made of multilayers sheets. In agreement with the current outcomes, Lombardo and coworkers [7] reported that the single-layer sheets (such as Zendura® and Duran®) were much stiffer than the multilayer sheets and generate higher initial forces. Nevertheless, they reported that single-layer materials showed much higher stress decay during a 24-hour period than multilayer counterparts. This is coherent with our findings for the one multilayered aligner material we tested, especially for in-/extrusive forces (Fig. 2) and for mesiodistal moments (Fig. 4). Nonetheless, as our investigated materials only contained one multilayered aligner material (i.e., Invisalign®), our study alone does not allow to attribute this behavior to this material itself or to the multilayered materials in general, but this finding needs further extensive research in future studies.

The mechanical properties of thermoplastic materials can be influenced by their molecular and crystal structures. While Essix ACE® (PET) and Duran® (PET-G) are amorphous plastics [36], Zendura® (TPU) is semicrystalline [37]. Under deformation, PET experiences strain-induced crystallization, but this happens significantly only when the material is exposed to elevated strains [36]. However, in the current study, only low deformation levels (0.2 mm) were applied; hence, no significant differences in mechanical characteristics were observed among these three materials after undergoing thermomechanical aging. Moreover, materials with higher glass transition temperature (Tg) exhibit greater mechanical stability against aging. The Tg values of the three materials are relatively similar, ranging from 75 to 80 °C [37], which are higher than the temperatures experienced during our thermocycling (5–55 °C).

The primary limitation of the current experimental study is the absence of a PDL, which may have led to the recording of higher force values. However, this factor can be effectively mitigated since we were comparing the behavior of different materials under identical conditions. Furthermore, significant alterations in the polymeric composition and the mechanical characteristics of the aligner could arise due to fluctuations in the oral cavity’s pH and the complex interaction with enzymes, ions, bacterial byproducts, as well as ingested substances. These factors were not accounted for in the study. Also, the OMSS records the total resultant force, lacking insights into force distribution across the tooth’s surface. Therefore, further research will be conducted to explore the variations in force distribution among different aligner materials.

## Conclusion

Studying the forces/moments generated by orthodontic aligners made of different thermoplastic materials showed the following:

- The force and moment magnitudes produced by aligners were significantly influenced by their material composition, with multilayer materials yielding lower forces and moments compared to their single-layer counterparts.
- The process of thermomechanical aging of aligners resulted in a prominent decrease in force during the initial 48 h, with almost no substantial additional decline from day 2 to day 14.
- Variability in post-aging force decay was evident across different materials, and the aging impact was less pronounced in multilayer materials compared to single-layer ones.
- Mechanical loading of aligners due to tooth malalignment and contact triggered cold hardening and subsequent increased material stiffness, consequently leading to an increase in the generated force.
- The force and moment generation by aligners on teeth was apparently influenced by the direction of the tooth movement.

## Data Availability

Data will be made available on request.
